# Small intestinal bleeding prediction by spectral reconstruction through band selection

**DOI:** 10.1117/1.JBO.30.3.036004

**Published:** 2025-03-19

**Authors:** Hsin-Yu Kuo, Riya Karmakar, Arvind Mukundan, Chu-Kuang Chou, Tsung-Hsien Chen, Chien-Wei Huang, Kai-Yao Yang, Hsiang-Chen Wang

**Affiliations:** a National Cheng Kung University Hospital, National Cheng Kung University, College of Medicine, Department of Internal Medicine, Tainan City, Taiwan; b National Chung Cheng University, Department of Mechanical Engineering, Chiayi, Taiwan; c Ditmanson Medical Foundation Chia-Yi Christian Hospital, Division of Gastroenterology and Hepatology, Department of Internal Medicine, Chiayi, Taiwan; d Ditmanson Medical Foundation Chia-Yi Christian Hospital, Obesity Center, Chiayi, Taiwan; e Ditmanson Medical Foundation Chia-Yi Christian Hospital, Department of Internal Medicine, Chiayi, Taiwan; f Kaohsiung Armed Forces General Hospital, Department of Gastroenterology, Kaohsiung, Taiwan; g Tajen University, Department of Nursing, Pingtung County, Taiwan; h Hitspectra Intelligent Technology Co., Ltd., Director of Technology Development, Kaohsiung, Taiwan; i Buddhist Tzu Chi Medical Foundation, Dalin Tzu Chi Hospital, Department of Medical Research, Chiayi, Taiwan

**Keywords:** hyperspectral imaging, wireless capsule endoscope, white-light imaging, narrow band imaging, convolutional neural network, visual geometry group-16, small intestine

## Abstract

**Significance:**

The identification of gastrointestinal bleeding holds significant importance in wireless capsule endoscopy examinations, primarily because bleeding is the most prevalent anomaly within the gastrointestinal tract. Moreover, gastrointestinal bleeding serves as a crucial indicator or manifestation of various other gastrointestinal disorders, including ulcers, polyps, tumors, and Crohn’s disease. Gastrointestinal bleeding may be classified into two categories: active bleeding, which refers to the presence of continuing bleeding, and inactive bleeding, which can potentially manifest in any region of the gastrointestinal system. Currently, medical professionals diagnose gastrointestinal bleeding mostly by examining complete wireless capsule endoscopy images. This approach is known to be demanding in terms of labor and time.

**Aim:**

This research used white-light images (WLIs) obtained from 100 patients using the PillCam™ SB 3 capsule endoscope to identify and label the areas of bleeding seen in the WLIs.

**Approach:**

A total of 152 photographs depicting bleeding and 182 images depicting non-bleeding were selected for analysis. In addition, hyperspectral imaging was used to transform WLI into hyperspectral images using spectral reconstruction through band selection. These images were then categorized into WLIs and hyperspectral images. The training set consisted of seven datasets, each including six spectra. These datasets were used to train the Visual Geometry Group-16 (VGG-16) model, which was developed using a convolutional neural network. Subsequently, the model was tested, and its diagnostic accuracy was assessed.

**Results:**

The accuracy rates for the respective measures are 83.1%, 65.8%, 66.2%, 72.2%, 73.7%, and 88%. The respective precision values are 78.5%, 47.5%, 30.6%, 59.5%, 77.7%, and 80.2%. The recall rates for the relevant data points are 83.3%, 67.9%, 86%, 74.2%, 68.6%, and 92.4%. The initial dataset comprises an image captured under white-light conditions, whereas the final dataset is the most refined spectral picture data.

**Conclusions:**

The findings suggest that employing spectral imaging within the wavelength range of 405 to 415 nm can enhance the accuracy of detecting small intestinal bleeding.

## 1 Introduction

The small intestine is primarily composed of three distinct sections: the duodenum, the jejunum, and the ileum. On average, the small intestine has a length of ∼6 to 7 m.^1^^–^^3^ The part of the digestive tract that spans from the posterior region of the stomach to the proximal end of the large intestine is the longest segment. The gastrointestinal tract serves as the primary organ responsible for the processes of food digestion and absorption. Although the prevalence of small intestinal disorders is very low,^4^^,^^5^ instances may arise where gastroscopy and colonoscopy fail to provide a definitive diagnosis for unexplained stomach pain,^6^ potentially indicating an underlying issue inside the small intestine.^7^ The process of diagnosis often entails a significant time investment, which subsequently leads to delays in the initiation of therapy. The occurrence of small bowel disease poses significant challenges^8^ for both patients and professionals, particularly in terms of diagnosis and treatment. The peak incidence of smooth muscle tumors in the small intestine in both male and female patients is between the ages of 50 and 59.^9^ The root cause of small intestinal bleeding lesions is contingent upon the patient’s age rather than their gender or race. Small intestinal hemorrhage is predominantly attributed to vasodilation in the small intestine.^10^^,^^11^ Several risk factors have been identified for the occurrence of vasodilation, including advancing age, the existence of aortic stenosis, chronic renal failure, utilization of a left ventricular assist device, and several genetic abnormalities.^12^ The utilization of wireless capsule endoscopy is of utmost importance in the identification of gastrointestinal bleeding. Gastrointestinal bleeding is the most frequent anomaly in the gastrointestinal tract.^13^^,^^14^ It serves as a significant indicator or concomitant manifestation of various gastrointestinal pathologies, including ulcers, polyps, tumors, and signs of Crohn’s disease.^15^^,^^16^ Gastrointestinal bleeding can be categorized into two distinct types: active bleeding, which refers to the presence of ongoing bleeding, and inactive bleeding, which has the potential to manifest in any region along the gastrointestinal tract. Currently, the identification of gastrointestinal bleeding primarily relies on the visual examination of complete wireless capsule endoscopy images^17^ by medical professionals. This approach is highly demanding in terms of labor and time.

Endoscopy remains the preeminent diagnostic modality for the identification of gastrointestinal disorders.^18^^–^^22^ Nevertheless, conventional endoscopy continues to exhibit certain limitations in a therapeutic setting. The endoscope hose and accompanying equipment are introduced to a significant depth within the gastrointestinal wall.^23^^,^^24^ Comprehensive treatment entails significant time investment^25^ and induces patient discomfort. Consequently, prioritizing capsule endoscopy as the primary approach for conducting intestinal examinations is advisable. In cases that require endoscopic assessment and intervention, various deep enteroscopy techniques may be employed. In the absence of any contraindications, conducting capsule endoscopy prior to deep enteroscopy is advisable. The application of computerized techniques in the detection of gastrointestinal bleeding in wireless capsule endoscopy images has garnered significant interest as a means of alleviating the burden on physicians.^4^^,^^26^^–^^29^ The literature study demonstrates that prevailing approaches utilizing shallow network architectures typically commence with a manually predetermined feature extraction phase, followed by an independent training procedure for the classifier. During the initial phase, many attributes such as color, texture, statistical information, and distinctive characteristics^30^ are manually retrieved from raw images obtained through wireless capsule endoscopy. The feature vectors that are created are utilized to train binary or discrete classifiers. The presence of hemorrhaging in the small intestine, a complicated medical condition that is notorious for its evasive nature, poses a substantial diagnostic challenge for medical professionals.^31^ Determining the cause of the bleeding accurately and quickly is crucial to deliver the proper treatment and improve the patient’s overall prognosis. Conventional diagnostic methods,^32^ such as endoscopy and radiographic imaging, have some shortcomings in precisely locating the source of bleeding in the small intestine. As a result, patients often must wait longer for treatment, thus worsening their pain and discomfort. The field of medical imaging has made significant strides in recent years, which has resulted in the development of creative solutions to this complex clinical dilemma.

The purpose of this research is to investigate the expanding field of hyperspectral imaging technology and its significant potential for accurately predicting the precise location of small intestinal hemorrhage. Our goal is to provide insight into the potentially disruptive implications of this technology in the field of gastrointestinal medicine by performing a comprehensive examination of the underlying principles, methods, and current advances. To achieve this goal, we will first undertake such an examination. Our goal is to lay a foundation for future research and development in this fascinating and important area of medicine by analyzing the challenges associated with the detection of small intestine hemorrhage and emphasizing the benefits of spectral reconstruction through band selection.

## 2 Materials and Methodology

**Fig. 1:**
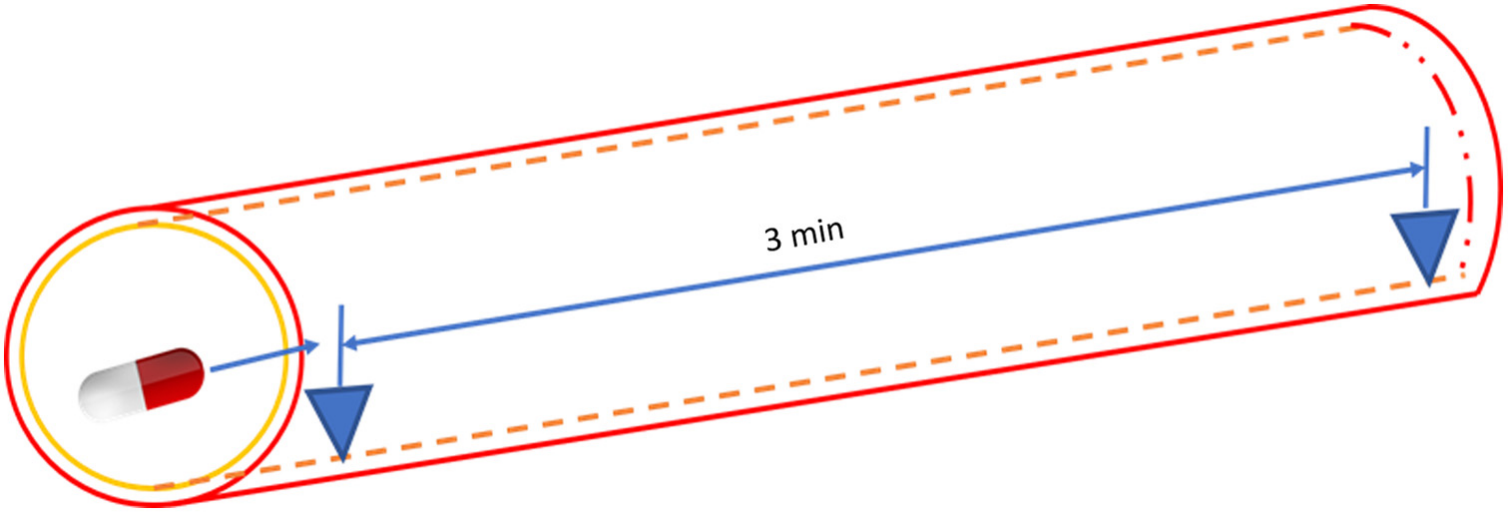
Image taken before blood spots are taken out.

**Fig. 2:**
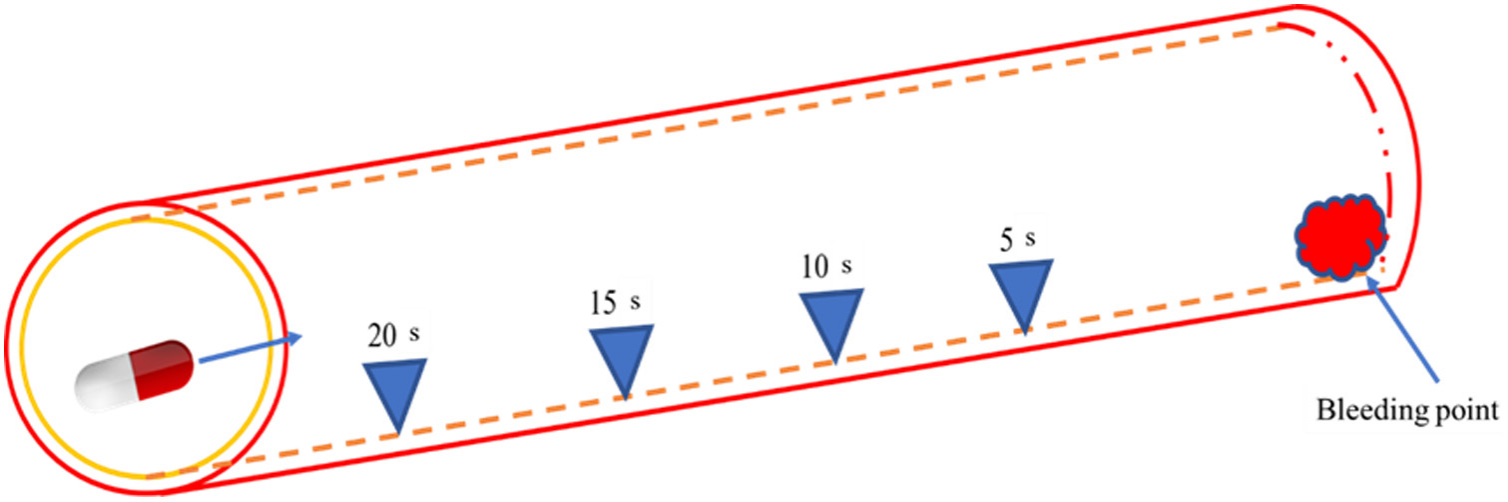
Image taken in the absence of hemorrhage.

In this study, we collaborated with the Department of Gastroenterology and Hepatobiliary Surgery at National Cheng Kung University, which provided white-light images (WLI) data captured using the PillCam™ SB 3^33^^–^^36^ capsule endoscope. Subsequently, the hemorrhage location identified during the acquisition of the WLIs was labeled. The dataset consisted of a total of 1336 photos, comprising 608 photographs depicting hemorrhage and 728 images without hemorrhage. The dataset comprises images from 100 patients who underwent routine diagnostic procedures. The study protocol and informed consent form were approved by the Medical Ethics Review Board of the National Cheng Kung University School of Medicine Hospital (IRB No. A-ER-111-145). All participants provided informed consent, and the study was conducted in accordance with relevant regulations and ethical guidelines. The experimental design and process were reviewed by the Medical Ethics Review Committee, ensuring compliance with ethical standards. As the study utilized pre-existing clinical data for analysis, it did not involve additional experimental interventions. This ethical oversight ensures the integrity and transparency of the study, supporting the validity of the results presented. Notably, the dataset did not include any low-quality images resulting from blurring, defocus, mucus, or poor air blowing. The procedure for evaluating hemorrhagic and non-bleeding segments is discussed comprehensively below. In this research, the images preceding the occurrence of hemorrhage were segmented and categorized into three time intervals: 5, 10, and 15 s prior to the hemorrhagic event, as depicted in Fig. 1. The classification process involves identifying the distinctive characteristics of the image during spectralization. For the non-bleeding section, 20 images were selected from the capsule images of patients with small intestinal bleeding, where no bleeding is observed for a continuous duration of 3 min. Figure 2 displays a picture depicting the absence of hemorrhage.

**Fig. 3:**
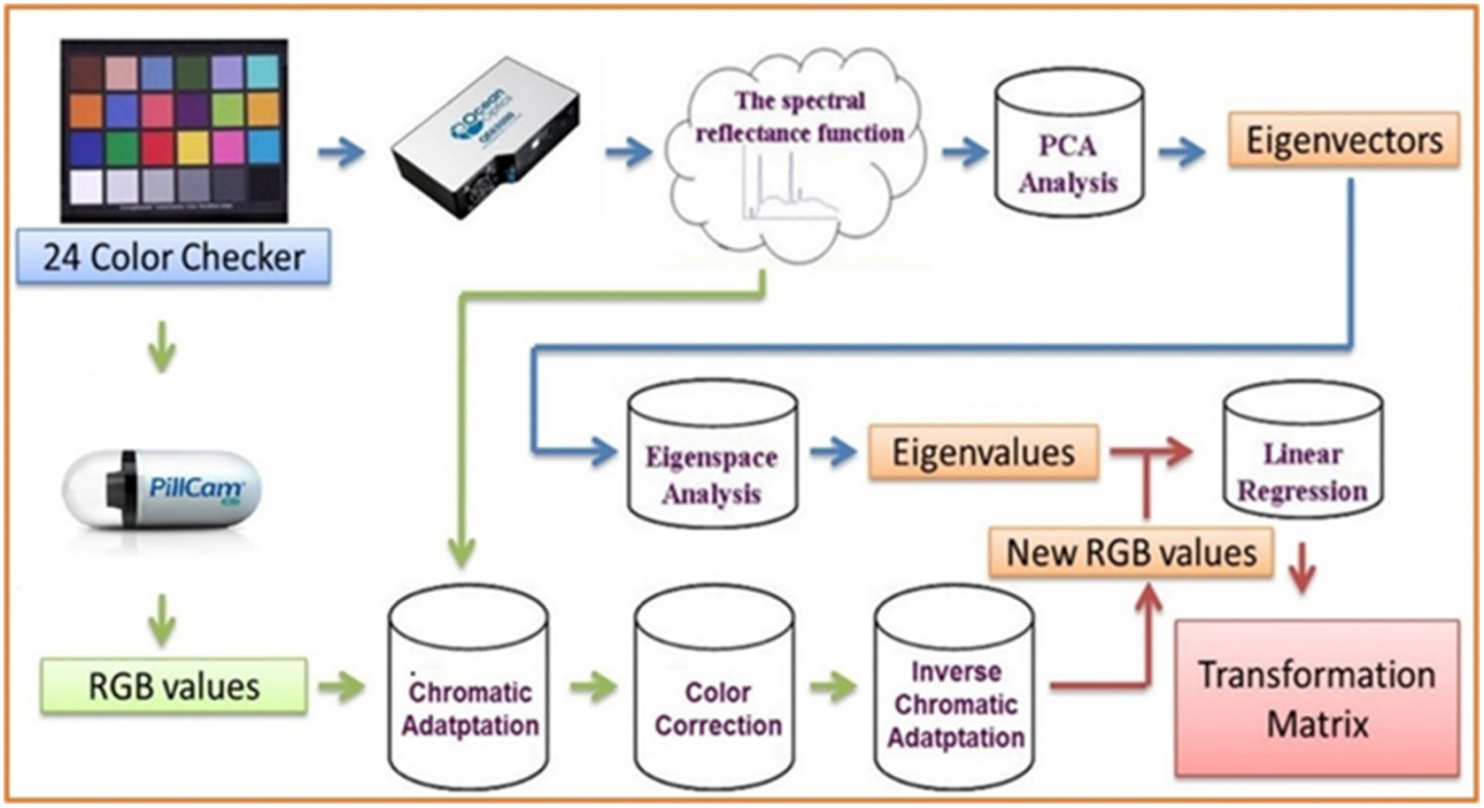
Spectral reconstruction through band selection conversion construction flowchart, using standard 24 color blocks (X-Rite Classic, 24 Color Checkers) as the common target object for endoscope and spectrometer spectrum conversion, converting endoscope images into 401 bands of visible spectral information.

The initial step is the translation of minor intestinal hemorrhage captured by the capsule endoscope into 401 bands of visible-light spectrum information (ranging from 380 to 780 nm) using spectral reconstruction through band selection. The procedure of converting the visual spectrum is depicted in Fig. 3. Spectrometers, namely, the Ocean Optics-QE65000 model, and a WLI endoscope are employed to measure the spectrum and capture photos of 24 color blocks. The research developed a spectral reconstruction through a band selection mechanism capable of transforming an red, green, blue (RGB) or a WLI captured from an endoscope into a HSI as depicted in Fig. 3. First, it is necessary to establish the correlation between the WLI image and the spectrometer for various colors. The Macbeth Color Checker (X-Rite Classic) is specifically marked as the set of colors to be used for calibration purposes. This tool comprises 24 squares displaying various color samples typically encountered in nature, including hues such as red, green, blue, cyan, magenta, and yellow, with six tons of gray. X-Rite has gained popularity as a preferred option for color calibration in recent years. The endoscopic camera was predominantly employed to collect photos that accurately represented the colors of the X-Rite board, also known as the target. The image consisting of 24 colors was converted to the CIE 1931 XYZ color space. The endoscopic images were saved in JPEG format using the standard RGB (sRGB) color scheme. The R, G, and B values of the sRGB color space, ranging from 0 to 255, were first converted to a narrower range between 0 and 1. Subsequently, the gamma function was employed to convert the reduced sRGB values into linearized RGB values. A translation matrix was employed to convert the linearized RGB values into the CIE 1931 color space and depict the numerical relationship between the wavelengths in the visible spectrum and the perceived colors in nature. On the other hand, images taken with an endoscope can be influenced by non-linear response, dark current, incorrect color separation, or color distortion. Subsequently, the CIE XYZ tristimulus values are derived by converting these normalized RGB values into XYZ values as XYZ camera. This conversion is facilitated through the application of a specific transformation equation as shown in Eq. (1).

$$[\begin{array}{c} X\\ Y\\ Z \end{array}]=[M_{A}][T][\begin{array}{c} f(R_{\mathrm{sRGB}})\\ f(G_{\mathrm{sRGB}})\\ f(B_{\mathrm{sRGB}}) \end{array}]\times 100,\;0\leq \begin{array}{c} R_{\mathrm{sRGB}}\\ G_{\mathrm{sRGB}}\\ B_{\mathrm{sRGB}} \end{array}\leq 1,$$ (1)

where $0\leq R_{\mathrm{sRGB}}$, $G_{\mathrm{sRGB}}$, and $B_{\mathrm{sRGB}}\leq 1$. Here, $R_{\mathrm{sRGB}}$, $G_{\mathrm{sRGB}}$, and $B_{\mathrm{sRGB}}$ denote the three color channels of the sRGB image. These channel values are linearized through a function defined by gamma correction. For sRGB images, this correction can be approximated by a power function with an exponent of 2.4, incorporating a linear segment for lower values to account for the non-linear sensitivity of human vision at these intensities. The equation for removing gamma correction, or linearization, is defined in Eq. (2).

$$[T]=[\begin{array}{ccc} 0.4104 & 0.3576 & 0.3576\\ 0.2126 & 0.7152 & 0.0722\\ 0.0193 & 0.1192 & 0.9505 \end{array}],$$ (2)

$$f(n)=\{\begin{array}{ll} {\left(\frac{n+0.055}{1.055}\right)}^{2.4}, & n>0.04045\\ (\frac{n}{12.92}), & \text{otherwise} \end{array},$$ (3)

$$[M_{A}]=[\begin{array}{ccc} X_{SW}/X_{CW} & 0 & 0\\ 0 & Y_{SW}/Y_{CW} & 0\\ 0 & 0 & Z_{SW}/Z_{CW} \end{array}].$$ (4)

Converting the obtained spectral data R(λ) (380∼780  nm, 1 nm) into the XYZ color gamut space requires the light source spectrum S(λ) of the ophthalmoscope hyperspectral system and the XYZ color matching function. Through Eqs. (5)–(7), the spectral data are converted into the XYZ values as $XYZ_{\text{Spectrometer}}$. The brightness ratios are given in Eq. (8).

$$X=k\int_{400\;\mathrm{nm}}^{700\;\mathrm{nm}}S(\lambda )R(\lambda )\overset{¯}{x}(\lambda )d\lambda ,$$ (5)

$$Y=k\int_{400\;\mathrm{nm}}^{700\;\mathrm{nm}}S(\lambda )R(\lambda )\overset{¯}{y}(\lambda )d\lambda ,$$ (6)

$$Z=k\int_{400\;\mathrm{nm}}^{700\;\mathrm{nm}}S(\lambda )R(\lambda )\overset{¯}{z}(\lambda )d\lambda ,$$ (7)

$$k=\frac{100}{\int_{400\;\mathrm{nm}}^{700\;\mathrm{nm}}S(\lambda )\overset{¯}{y}(\lambda )d\lambda },$$ (8)

where S(λ) is the light source spectrum in XYZ color gamut space, and $\overset{¯}{x}(\lambda )$, $\overset{¯}{y}(\lambda )$, and $\overset{¯}{z}(\lambda )$ are the XYZ values of the color-matching function. The spectral data signal R(λ) from the spectrometer is converted to the XYZ color space. Equation (9) was employed to find the color correlation between the spectrophotometer and the camera. Equation (10) was used to calculate the updated X, Y, and Z values ($XYZ_{\text{Correct}}$) after error correction.

$$[C]=[XYZ_{\text{Spectrum}}]\times \mathrm{pinv}([V]),$$ (9)

$$[XYZ_{\text{Correct}}]=[C]\times [V].$$ (10)

The camera’s nonlinear response can be adjusted using a third-order equation, and the variable that represents the modification of the nonlinear response is defined as $V_{\text{Non-linear}}$.

$$V_{\text{Non}-\text{linear}}={\left[\begin{array}{cccccccccc} X^{3} & Y^{3} & Z^{3} & X^{2} & Y^{2} & Y^{2} & X & Y & Z & 1 \end{array}\right]}^{T}.$$ (11)

In the dark current component of an imaging device, the dark current remains constant regardless of the amount of light it receives. Therefore, a specific value is assigned to reflect the contribution of the dark current, and the variable for modifying the dark current is established appropriately as

$$V_{\text{Dark}}=[a].$$ (12)

The variable matrix V is obtained by standardizing the product of $V_{\text{Color}}$ and $V_{\text{Non-linear}}$, together with the inclusion of $V_{\text{Dark}}$. To avoid excessive correction, the standardization is restricted to the third order,

$$V_{\text{Color}}={\left[\begin{array}{ccccccc} XYZ & XY & XZ & YZ & X & Y & Z \end{array}\right]}^{T},$$ (13)

$$V=[\begin{array}{c} X^{3}\;Y^{3}\;Z^{3}\;\\ X^{2}Y\;X^{2}Z\;Y^{2}Z\;\\ XY^{2}\;XZ^{2}\;YZ^{2}\;\\ XYZ\;X^{2}\;Y^{2}\;Y^{2}\;\\ XY\;XZ\;YZ\;X\;Y\;Z\;a \end{array}{]}^{T}.$$ (14)

Before using CIE DE2000 to calculate color differences, it is essential to translate $XYZ_{\text{Correct}}$ and $XYZ_{\text{Spectrum}}$ from the XYZ color system to the lab color space. The equation for conversion is as follows:

$$L^{*}=116f(\frac{Y}{Y_{n}})-16\;a^{*}=500[f(\frac{X}{X_{n}})-f(\frac{Y}{Y_{n}})]\;b^{*}=200[f(\frac{Y}{Y_{n}})-f(\frac{Z}{Z_{n}})],$$ (15)

$$f(n)=\{\begin{array}{ll} n^{\frac{1}{3}}, & n>0.008856\\ 7.787n+0.137931, & \text{otherwise} \end{array}.$$ (16)

The spectra were organized into a matrix, denoted as ($R(\lambda ){)}_{401\;\times \;24}$, where the columns represent the number of color checkers, and the rows correspond to the intensities of the wavelengths at 1 nm intervals. Twelve eigenvectors, identified as having the most significant contribution, were established as the basis for spectral calculation and organized into a matrix $(E{)}_{12\;\times \;401}$ by calculating the eigen system and utilizing principal component analysis (PCA). The corresponding eigenvalues of these six eigenvectors, denoted as $(\alpha {)}_{12\;\times \;24}$, were determined using Eq. (17).

$$[\alpha {]}^{T}=[R(\lambda {)}^{T}]\mathrm{pinv}[E].$$ (17)

The reflectance spectrum data were converted into XYZ values ($XYZ_{\text{Spectrum}}$), which were then normalized inside the XYZ color gamut space. The correction coefficient matrix C was derived by the process of multiple regression, specifically using Eq. (18). The reflectance spectrum data ($R_{\text{spectrum}}$) were utilized to calculate the transformation matrix (M) for the colors included in the X-Rite board. PCA was conducted on the $R_{\text{spectrum}}$ dataset to extract the six most significant principal components (PCs) and their corresponding eigenvectors. The six PCs were able to account for 99.64% of the information. The mean root mean square error (RMSE) of the 24 desired colors between $XYZ_{\text{Correct}}$ and $XYZ_{\text{Spectrum}}$ was 0.19, indicating that the difference is insignificant. Subsequently, a multiple regression analysis was conducted on variable M, and its correlation with the six significant principal components was examined. The six PCs were utilized for conducting a multivariate regression analysis of $XYZ_{\text{Correct}}$. In this study, the variable $V_{\text{Color}}$ was selected with great care due to its ability to encompass all possible combinations of the X, Y, and Z values.

$$[M]=[\text{Score}]\times \mathrm{pinv}([V_{\text{Color}}]).$$ (18)

The analog spectrum ($S_{\text{Spectrum}}$) was computed from $XYZ_{\text{Correct}}$ using Eq. (19). Next, $S_{\text{Spectrum}}$ was assessed in comparison with $R_{\text{spectrum}}$. The mean RMSE of the 24 color blocks was merely 0.056, whereas the average discrepancy in color between the acquired analog spectrum and the reflectance spectrum produced by the spectrometer was only 0.75. This discovery suggests that the colors derived from the reflectance spectrum closely matched the colors representing the observed values. Thus, by employing the aforementioned technique, it is possible to convert an RGB image captured by an endoscope into an HSI image.

$$[S_{\text{Spectrum}}{]}_{380∼780\;\mathrm{nm}}=[EV][M][V_{\text{Color}}].$$ (19)

**Fig. 4:**
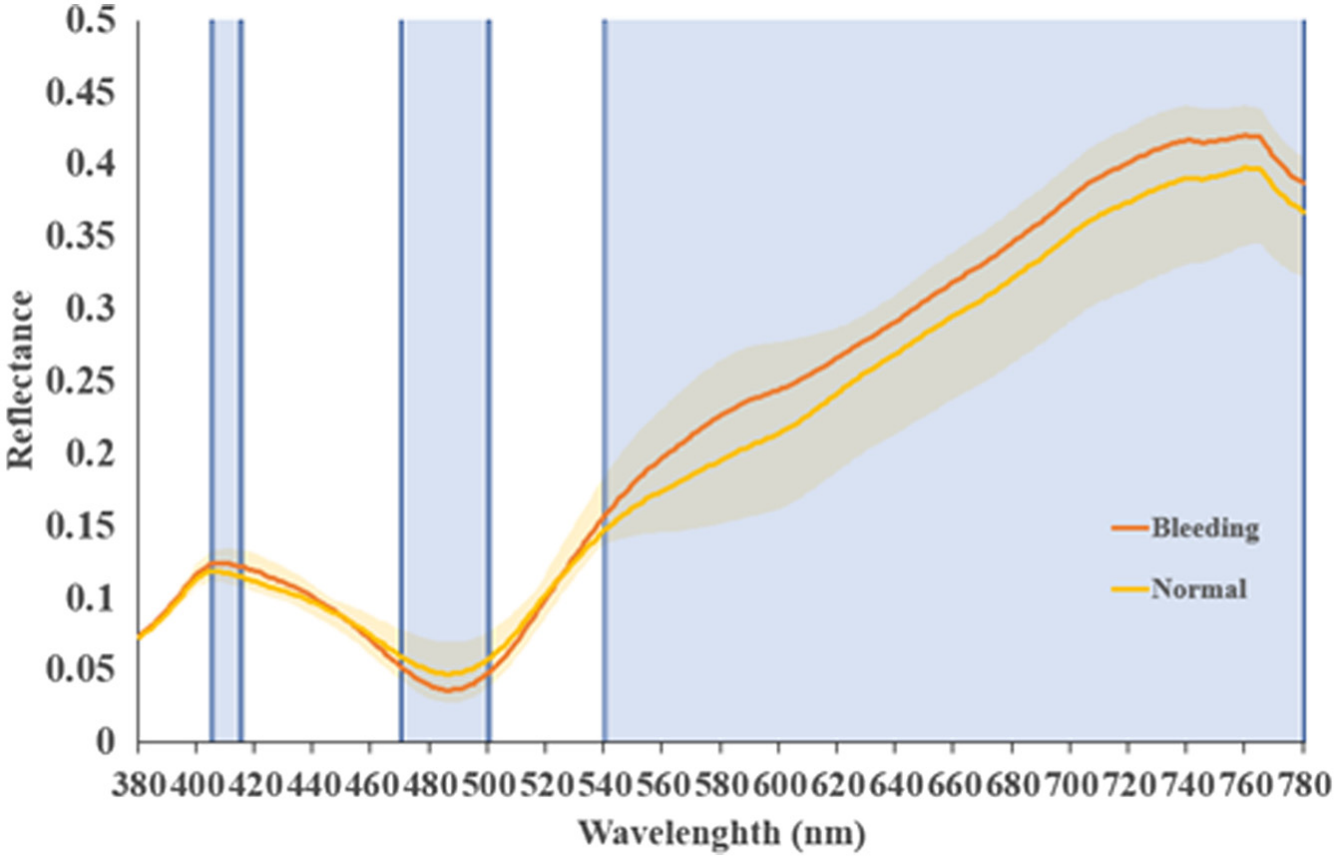
Spectrum distribution chart of the small intestine captured by endoscopy, distinguishing between bleeding and non-bleeding areas. Subsequently, after the observations were analyzed, a composite array is formed by combining spectral data within the ranges of 405 to 415, 470 to 500, and 540 to 780 nm.

This method yields a collection of spectrum data and RGB three-channel images. Subsequently, the two sets of data are processed to determine the disparity among them. The conversion matrix is employed to transform the acquired spectrum information into the visible-light band (380 to 780 nm) through spectral reconstruction through band selection. By marking positions, we analyze the spectral disparities between lesions and non-lesions, as depicted in Fig. 4. Specifically, we utilize spectral information ranging from 405 to 415, 470 to 500, and 540 to 780 nm, which are organized and combined into seven datasets that include WLIs for further utilization. The initial dataset comprises white-light data lacking any discernible spectrum. The extraction of additional datasets relies on the utilization of the separation spectrum range depicted in Fig. 4 to ascertain the presence of any bleeding. The second dataset comprises measurements within the wavelength range of 540 to 780 nm. This band was selected because it can achieve the highest resolution within the red-light spectrum. The third dataset corresponds to the 470 to 500 nm range. This band was selected because of its superior degree of separation within the green-light spectrum. The fourth dataset comprises a synthetic spectrum dataset spanning the wavelength range of 470 to 500 and 540 to 780 nm. This study investigates the potential integration of differences in the overall spectrum of red and green light. The fifth dataset comprises synthetic spectrum data ranging from 405 to 415, 470 to 500, and 540 to 780 nm, which helps in investigating the potential for synthesizing differences in spectrum synthesis across red, green, and blue light. The sixth dataset corresponds to the range of 405 to 415 nm. This particular band was selected because of its superior differentiation within the range of blue-light wavelengths. The eighth dataset consists of wavelengths ranging from 405 to 415 and 535 to 545 nm. The rationale behind selecting this band is rooted in its alignment with the spectral range commonly utilized in traditional narrow band imaging.

The Visual Geometry Group-16 (VGG-16) model is primarily designed for the purpose of feature extraction. The subject matter is discussed comprehensively in the initial section of the Supplementary Material. The architectural representation of VGG-16 consists of many convolutional neural networks, as previously discussed.

The VGG model is a prominent method in the field of image classification. It is designed to assign an entire image to a specific category without determining the precise position or coordinates of objects inside the image, unlike object detection. Object detection involves determining the precise location of an object inside an image. Hence, when the VGG model is used to identify unfamiliar images, the unknown image should have only a solitary object, whereas the background is ideally kept minimalistic.

VGGNet can be considered a more detailed version of the AlexNet architecture. The AlexNet design employs a convolutional kernel with a size of 7×7, whereas the VGGNet architecture utilizes a convolutional kernel with a size of 3×3, enabling the implementation of a more intricate and extensive convolutional neural network. Research has demonstrated that augmenting the depth of a convolutional neural network by incorporating additional hidden layers and weights can yield notable enhancements in recognition accuracy. The architectural design of VGGNet resembles that of AlexNet but with the incorporation of more hidden layers. This augmentation results in an expanded parameter adjustment range, hence leading to the final model parameters being approximately three times greater than those of AlexNet. The VGG model employs a dataset consisting of one million images for the purpose of training, afterward categorizing them into a thousand distinct classes. This paradigm has evolved into a widely applicable solution. If the image does not fall within the scope of these 1000 categories, then the input convolutional layer can be substituted and the middle layer can be used to extract features. This approach is commonly referred to as transfer learning. The VGG architecture consists of two variations, namely, VGG-16 and VGG-19. VGG-16 is composed of 13 convolutional layers and 3 fully connected layers, whereas VGG-19 has 16 convolutional layers and 3 fully connected layers.

## 3 Results

**Fig. 5:**
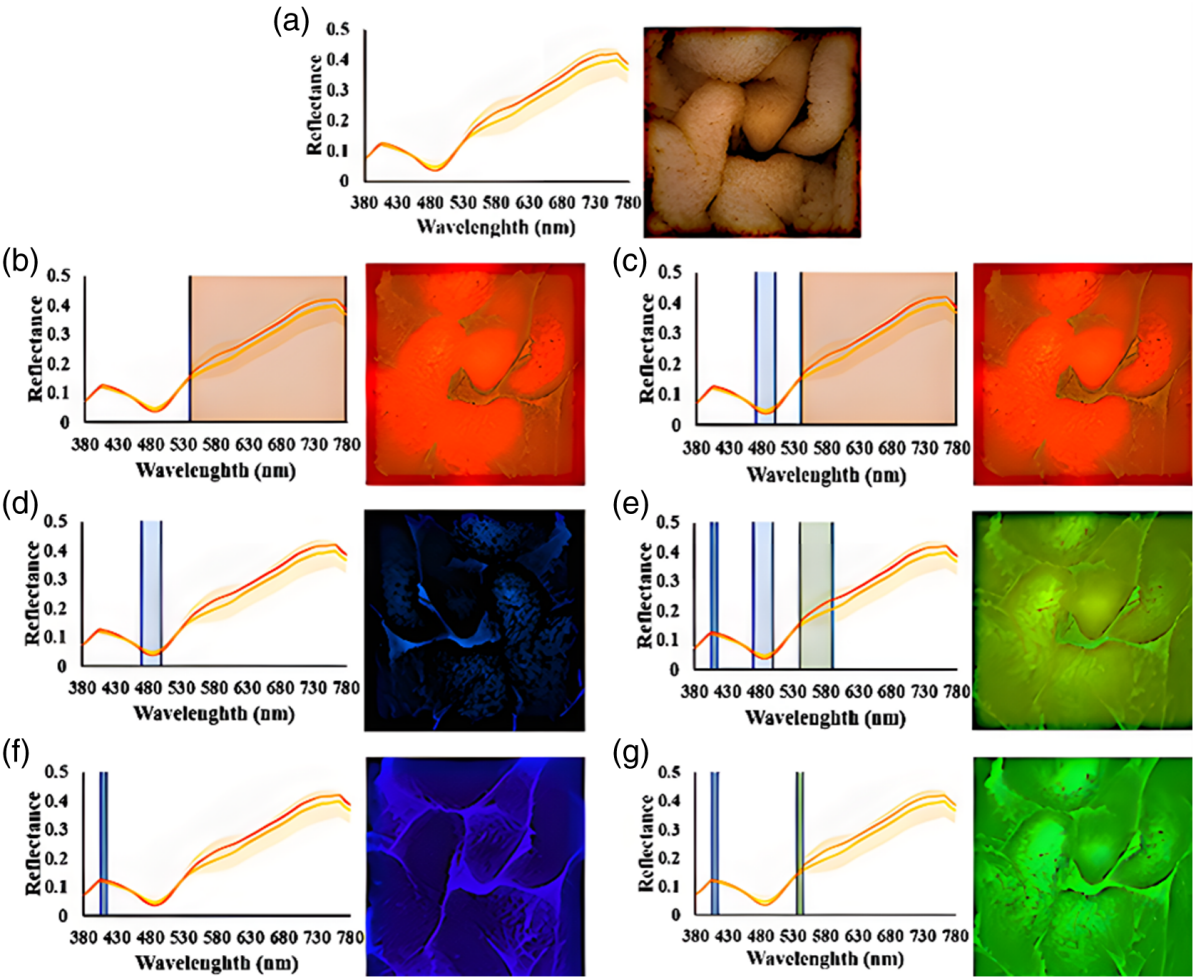
Diagrams depicting different spectra for data collection. (a) Spectrum selection range of the white light picture on the left and schematic design for image reproduction on the right. (b) Spectrum selection of 540 to 780 nm on the left and the corresponding image reproduction on the right. (c) Spectrum selection range of 540 to 780 and 470 to 500 nm on the left and the picture reproduction diagram on the right. (d) Spectrum selection of 470 to 500 nm on the left and the picture reproduction diagram on the right. (e) Spectrum selection range of 470 to 500, 405 to 415, and 540 to 590 nm on the left and the schematic on the right. (f) Spectrum selection range of 405 to 415 nm on the left and the corresponding image reproduction on the right. (g) Spectrum of 405 to 415 and 535 to 545 nm on the left and the corresponding image reproduction on the right.

This study utilized capsule endoscopic images from a sample of 100 patients to develop prediction models for chromaticity (RGB) and hyperspectral images to distinguish between hemorrhage and non-bleeding cases. Three spectral ranges were extracted and combined to create a total of seven models, which were then evaluated. Figure 5(a) displays the prediction results of the white-light esophageal cancer image detection model and the white-light hyperspectral esophageal cancer image detection model. The left side of Fig. 5(a) presents the spectrum selection range of the WLI, whereas the right side depicts a schematic of image reproduction. The left side of Fig. 5(b) illustrates the spectrum selection range of 540 to 780 nm, whereas the right side depicts a schematic of image reproduction. Similarly, the left side of Fig. 5(c) displays the spectrum selection range of 540 to 780 and 470 to 500 (nm), with the right side showing a schematic of image reproduction. The left side of Fig. 5(d) presents the spectrum selection range of 470 to 500 nm, accompanied by a schematic of image reproduction on the right side. Finally, the left side of Fig. 5(e) pertains to the range of 470 to 500 nm. The spectral selection range of 405 to 415 and 540 to 590 nm is depicted in the schematic of picture reproduction. The left side of Fig. 5(f) shows the spectrum selection range of 405 to 415 nm, whereas the right side represents the image reproduction. The schematic in Fig. 5(g) illustrates the spectrum selection range of 405 to 415 and 535 to 545 nm on the left side, whereas the right side depicts a schematic of image reproduction.

The results of the WLI small intestinal bleeding image detection model indicate that out of a total of 121 hemorrhage photos, 95 were accurately predicted. Similarly, out of 145 non-bleeding images, 126 were successfully predicted. Calculations determine that the overall accuracy rate is 83.1%. The bleeding sensitivity is 83.3%, whereas the non-bleeding sensitivity is 82.9%. The confusion matrix for the detection model of small intestinal bleeding images in the wavelength range of 540 to 780 nm consists of a total of 121 bleeding photos, of which 57 predictions were accurate. In addition, the matrix includes 145 non-bleeding images. A total of 118 predictions in the photos were accurately identified, resulting in an overall accuracy rate of 65.8%. The sensitivity for detecting bleeding in the images was 67.9%, whereas the sensitivity for detecting non-bleeding cases was 64.8%. Small intestinal bleeding image detection was performed within the wavelength range of 470 to 500 and 540 to 780 nm. Within the confusion matrix of the model, 37 out of 121 hemorrhage photos were accurately predicted, whereas 139 out of 145 non-bleeding images were successfully predicted. Computations indicate that the overall accuracy rate was 66.2%. The model for detecting small intestinal bleeding images at a wavelength range of 470 to 500 nm achieved a sensitivity of 86% for images with hemorrhage and 62.3% for images without hemorrhage. In the confusion matrix, out of the total 121 hemorrhage images, 72 were correctly predicted as hemorrhage, whereas 145 were correctly forecasted as no hemorrhage. A total of 120 predictions were accurate, with a total accuracy rate of 72.2%. The sensitivity for detecting blood was 74.2%, whereas the sensitivity for detecting the absence of bleeding was 71%. In addition, the wavelength ranges used in the analysis were 405 to 415, 470 to 500, and 540 to 590 nm. In the confusion matrix pertaining to the small intestinal bleeding image detection model, 94 out of a total of 121 bleeding photos were accurately predicted. Similarly, 102 out of a total of 145 non-bleeding images were correctly predicted. This result indicates an overall accuracy rate of 73.7%. The sensitivity for hemorrhage is 68.6%, whereas the sensitivity for non-bleeding is 79.1%. The confusion matrix of the small intestinal bleeding image detection model consists of values ranging from 405 to 415 nm. Out of a sample size of 121 bleeding photographs, a total of 97 predictions were accurate. Similarly, out of a sample size of 145 non-bleeding images, 137 predictions were accurate. The overall accuracy of the small intestinal bleeding image detection model was 88%. The sensitivity to bleeding was 92.4%, whereas the sensitivity to non-bleeding was 85.1%. The confusion matrix for the model, specifically for the wavelength range of 405 to 415 and 535 to 545 nm, revealed that out of a total of 121 bleeding images, 69 predictions were accurate. Similarly, out of 145 non-bleeding images, 113 predictions were correct. The overall accuracy was computed, obtaining a 68% sensitivity for both bleeding and non-bleeding.

## 4 Discussion

Table 1 presents the results of the evaluation standard. The data in the table show that several performance metrics of WLI, including accuracy, recall, specificity, and F1 score, exceed 80%. Figure 5(a) displays the original image on the right side. The objective of this study is to utilize spectral reconstruction through band selection to enhance the accuracy of predicting the relative location of small intestinal bleeding. This objective is achieved by conducting subsequent spectral analysis on the basis of WLI. Spectral images whose evaluation indices surpass those of WLI will be considered effective spectra for the detection of small intestinal bleeding.

**Table 1:** Results of model evaluation indicators for seven distinct datasets.

	Accuracy	Precision	Recall	Specificity	F1 score
WLI	0.831	0.785	0.833	0.829	0.809
540 to 780 nm	0.658	0.471	0.679	0.648	0.556
470 to 500 and 540 to 780 nm	0.662	0.306	0.860	0.623	0.451
470 to 500 nm	0.722	0.595	0.742	0.710	0.661
405 to 415, 470 to 500, and 540 to 590 nm	0.737	0.777	0.686	0.791	0.729
405 to 415 nm	0.880	0.802	0.924	0.851	0.858
405 to 415 and 535 to 545 nm	0.684	0.57	0.683	0.684	0.62

### 4.1 540 to 780 nm

The first spectrum encompasses the wavelength range of 540 to 780 nm. Figure 5(b) displays the replicated picture and the chosen band. The image graphically demonstrates the presence of several distortions inside the shown elements. Even though the learning rate and the number of training iterations were adjusted, the accuracy did not improve, but the loss was successfully decreased. This result is hypothesized to be due to the fact that, although a significant disparity in reflection intensity between 540 and 780 nm occurs within the spectrum selection, a similar situation arises across the entire spectrum. Consequently, the magnitude of reflection intensity change varies minimally. Therefore, this spectrum is unsuitable for application in small intestine imaging.

### 4.2 470 to 500 and 540 to 780 nm

The spectral range of the subsequent replicated image spans from 470 to 500 and 540 to 780 nm. The process of replicating the image and choosing the specific band is illustrated in Fig. 5(c). Comparable issues are observed within the wavelength range of 540 to 780 nm and a significant loss of information occurs, as indicated by a comparison of the 540 to 780 nm range to the level of detail perceivable by the naked eye. The data in Table 1 indicate that the recall rate for detecting bleeding in the small intestine within this particular band is 86%, surpassing the value obtained within the 540 to 780 nm wavelength range. Nevertheless, the precision and various other metrics are significantly lower than those of WLIs, thus requiring further study.

### 4.3 470 to 500 nm

To investigate the potential obscuration of small intestinal details by red light, we conducted an experiment involving the observation of spectra visible to the human eye. Specifically, we omitted the 540 to 780 nm wavelength range and replicated the image. Subsequently, we selected the band as depicted in Fig. 5(d). The spectral range of this band is limited to 470 to 500 nm. The findings in Table 1 show that the accuracy rate significantly increased to 72%. This improvement suggests that the exclusion of the red-light spectrum may provide more favorable outcomes in the context of learning. However, the accuracy rate remains lower than that associated with white light. Therefore, other light spectra need to be explored.

### 4.4 405 to 415, 470 to 500, and 540 to 590 nm

An examination of the typical spectral characteristics of hemorrhage and non-bleed cases shows distinct separations in the spectra within the wavelength ranges of 405 to 415, 470 to 500, and 540 to 780 nm. However, previous experimental findings suggest that incorporating the red-light spectrum into the reproduced image spectrum reduces the indicators. Consequently, for the 540- to 780-nm band, only the green-light spectrum within the range of 540 to 590 nm is utilized for image reproduction and selection. The bands are depicted in Fig. 5(e). The data presented in Table 1 show that the current accuracy rate of the wave band reached 74%, indicating a notable improvement. The imaging of the bleeding small intestine demonstrated enhanced outcomes, with greater accuracy and a comprehensive index F1 score of 100%. Incorporating the 405- to 415-nm band into the model significantly enhanced the curve, resulting in a comparatively high score of 73%. This finding suggests that this particular band contains a greater number of eigenvalues within the spectrum.

### 4.5 405 to 415 nm

On the basis of the aforementioned experimental data, the spectral range of 405 to 415 nm is postulated to exhibit a greater number of characteristic values. Consequently, the single band encompassing 405 to 415 nm within the blue band was used. The image reproduction and selection of bands are shown in Fig. 5(f). The findings in Table 1 show that the measurements within the 405- to 415-nm wavelength range achieved an accuracy of 88%. Furthermore, other data parameters improved, surpassing the evaluation criteria established by the wavelength index. Hence, this spectrum is considered effective in detecting minor intestinal hemorrhage in images.

### 4.6 405 to 415 and 535 to 545 nm

The 405- to 415-nm spectral band is within the narrow band (NB) image. Thus, exploring the utilization of the standard NB image band, namely, the range of 405 to 415 + 535 to 545 nm, is advantageous. Employing this approach may potentially enhance the image quality and subsequently allow an informed decision to be made regarding the selection of the band. As depicted in Fig. 5(g), according to the data in Table 1, the accuracy declined to 68%. Interference potentially occurred among the green-light spectrum—specifically, the range above 470 nm—and the blue-light spectrum—specifically, 415 nm—in the visualization of the small intestine.

The findings in Table 1 show that the evaluation index for the 405- to 415-nm band surpasses that of the remaining five spectra, hence exhibiting superior accuracy to WLIs. On the basis of scholarly literature, this band can be inferred to be a constituent of the NB image. The NB images were selected to facilitate the examination of microvessels inside surface mucosal tissue. The primary factor contributing to the absorption rate of hemorrhage at 415 nm is its relatively high magnitude, although the penetrating capability of blue light is comparatively weaker than that of red light. Surface tissue can absorb both light and green light and thus can be effectively utilized to enhance the visibility of microvascular tissue. The inclusion of the 535 to 545 nm band in the analysis may decrease the accuracy due to the obstruction caused by small intestinal villi to other spectra with greater penetration capabilities. Superfluous spectral data are introduced when the blue-light spectrum is combined with the green-light spectrum, which is not absorbed by the villus wall of the small intestine. The majority of the visual representations observed within the small intestine pertain to the small intestinal villi, which are anatomical structures characterized by the presence of villous capillaries. Small intestinal hemorrhage is predominantly attributed to aberrant growth of vascular tissue. Hence, atypical proliferation of vascular tissue occurs on the surface of the small intestine. The spectral range between 405 and 415 nm is advantageous in the identification of surface mucosal tissue lesions. Consequently, this spectral band can be used as a dataset to predict the location of a small intestinal bleeding model. This study employs a spectral conversion process using band selection to transform WLI into spectral data, focusing on evaluating specific spectral ranges. Unlike traditional hyperspectral imaging methods that analyze full-spectrum data, this approach targets narrow spectral bands for bleeding detection. Direct comparisons with state-of-the-art HSI methods may not be meaningful due to methodological differences in data acquisition and spectral processing. Our work establishes a foundation for enhancing imaging techniques through targeted spectral analysis.

## 5 Conclusion

Our research has demonstrated that spectral reconstruction through band selection technology significantly improves the rapid detection and accurate positioning of minor gastrointestinal hemorrhages in comparison with conventional WLIs. Utilizing the VGG-16 model, which has been created for analyzing hyperspectral information related to minor intestinal hemorrhage, our research derived valuable insights. Our evaluation index identified the 405- to 415-nm wavelength range as the most promising option for accurate lesion identification. Moreover, our model has an excellent average accuracy rate of 88%, indicating that we have made substantial progress in achieving precision. Although there is still room for improvement, this achievement highlights the significance of an in-depth examination of the spectral characteristics within this specific wavelength range. Our upcoming endeavors include the procurement of additional data to improve the model’s precision. We anticipate that the widespread application of this technology in our future endeavors will substantially improve the ability of endoscopists to recognize signs of bleeding. Incorporating spectral reconstruction through band selection into clinical practice can have a revolutionary effect on the field of gastrointestinal diagnostics, resulting in enhanced patient care and treatment outcomes. As the investigation of spectral reconstruction through band selection progresses, attaining greater precision and efficacy in the localization of bleeding becomes increasingly possible.

## Supplementary Material

10.1117/1.JBO.30.3.036004.s01

## Data Availability

The data presented in this study are available in this article upon the request to the corresponding authors.

## References

[1] Jadhav S. S. et al., “Length of small intestine in formalin fixed adult human cadavers,” Int. J. Health Sci. Res. 5, 135–139 (2015).

[r2] Romanes G., Cunningham’s Manual of Practical Anatomy, Vol. 3, Oxford University Press (1986).

[3] Hounnou G. et al., “Anatomical study of the length of the human intestine,” Surg. Radiol. Anat. 24, 290 (2002). 10.1007/s00276-002-0057-y 12497219

[4] Alizadeh M. et al., “Segmentation of small bowel tumors in wireless capsule endoscopy using level set method,” in IEEE 27th Int. Symp. on Comput.-Based Med. Syst., IEEE, pp. 562–563 (2014). 10.1109/CBMS.2014.140

[5] Ghosh T. et al., “A statistical feature based novel method to detect bleeding in wireless capsule endoscopy images,” in Int. Conf. Inf., Electron. & Vision (ICIEV), IEEE, pp. 1–4 (2014). 10.1109/ICIEV.2014.6850777

[6] Vieira P. et al., “Segmentation of small bowel tumor tissue in capsule endoscopy images by using the MAP algorithm,” in Annu. Int. Conf. IEEE Eng. in Med. and Biol. Soc., IEEE, pp. 4010–4013 (2012). 10.1109/EMBC.2012.6346846 23366807

[7] Boardman L., “Heritable colorectal cancer syndromes: recognition and preventive management,” J. Clin. Oncol. 31, 1107–1131 (2002). 10.1016/s0889-8553(02)00049-3 12489281

[8] Chalopin C. et al., “Intraoperative imaging for procedures of the gastrointestinal tract,” in Innovative Endoscopic and Surgical Technology in the GI Tract, Horgan S. Fuchs K. H., Eds., pp. 365–379, Springer, Cham (2021).

[9] Blanchard D. K. et al., “Tumors of the small intestine,” World J. Surg. 24, 421–429 (2000). 10.1007/s002689910067 10706914

[10] Leitner R. et al., “High-sensitivity hyperspectral imager for biomedical video diagnostic applications,” Proc. SPIE 7674, 76740E (2010). 10.1117/12.849442

[11] Li Q. et al., “Review of spectral imaging technology in biomedical engineering: achievements and challenges,” J. Biomed. Opt. 18, 100901 (2013). 10.1117/1.JBO.18.10.100901 24114019

[12] Gerson L. B. et al., “ACG clinical guideline: diagnosis and management of small bowel bleeding,” Amer. J. Gastroenterol. 110, 1265–1287 (2015). 10.1038/ajg.2015.246 26303132

[13] Gopalakrishnan K. et al., “Deep convolutional neural networks with transfer learning for computer vision-based data-driven pavement distress detection,” Constr. Build. Mater. 157, 322–330 (2017). 10.1016/j.conbuildmat.2017.09.110

[14] Grigoroiu A. et al., “Deep learning applied to hyperspectral endoscopy for online spectral classification,” Sci. Rep. 10, 3947 (2020). 10.1038/s41598-020-60574-6 32127600 PMC7054302

[15] Song J. H. et al., “The etiology of potential small-bowel bleeding depending on patient’s age and gender,” United Eur. Gastroenterol. J. 6, 1169–1178 (2018). 10.1177/2050640618797841 PMC6169048 30288279

[16] Sato D. et al., “Distinction of surgically resected gastrointestinal stromal tumor by near-infrared hyperspectral imaging,” Sci. Rep. 10, 21852 (2020). 10.1038/s41598-020-79021-7 33318595 PMC7736345

[17] Jansen-Winkeln B. et al., “Hyperspectral imaging of gastrointestinal anastomoses,” Der Chirurg. 89, 717–725 (2018). 10.1007/s00104-018-0633-2 29637244

[18] Katsinelos P. et al., “The role of capsule endoscopy in the evaluation and treatment of obscure-overt gastrointestinal bleeding during daily clinical practice: a prospective multicenter study,” Scand. J. Gastroenterol. 49, 862–870 (2014). 10.3109/00365521.2014.889209 24940823

[r19] Khan M. J. et al., “Modern trends in hyperspectral image analysis: a review,” IEEE Access 6, 14118–14129 (2018). 10.1109/ACCESS.2018.2812999

[r20] Fang Y.-J. et al., “Identification of early esophageal cancer by semantic segmentation,” J. Pers. Med. 12, 1204 (2022). 10.3390/jpm12081204 35893299 PMC9331549

[r21] Huang H.-Y., et al., “Classification of skin cancer using novel hyperspectral imaging engineering via YOLOv5,” J. Clin. Med. 12, 1134 (2023). 10.3390/jcm12031134 36769781 PMC9918106

[22] Liao W.-C. et al., “Systematic meta-analysis of computer-aided detection to detect early esophageal cancer using hyperspectral imaging,” Biomed. Opt. Express 14, 4383–4405 (2023). 10.1364/BOE.492635 37799695 PMC10549751

[23] Parsi M. A. Burke C., “Utility of capsule endoscopy in Peutz-Jeghers syndrome,” Gastrointest. Endosc. Clin. N. Amer. 14, 159–167 (2004). 10.1016/j.giec.2003.10.012 15062389

[24] Raj A. et al., “Enhanced vascular features in porcine gastrointestinal endoscopy using multispectral imaging,” in 44th Annu. Int. Conf. IEEE Eng. in Med. & Biol. Soc. (EMBC), IEEE, pp. 2228–2231 (2022). 10.1109/EMBC48229.2022.9871634 36086222

[25] Kim P. et al., “Convolutional neural network,” MATLAB Deep Learning, pp. 121–147, Apress, Berkeley, California (2017).

[26] Barbosa D. J. et al., “Automatic detection of small bowel tumors in capsule endoscopy based on color curvelet covariance statistical texture descriptors,” in Annu. Int. Conf. IEEE Eng. in Med. and Biol. Soc., IEEE, pp. 6683–6686 (2009). 10.1109/IEMBS.2009.5334013 19964706

[r27] Kodogiannis V. et al., “Neuro-fuzzy classification system for wireless-capsule endoscopic images,” Int. J. Electr. Comput. Syst. Eng. 2, 55–63 (2008).

[r28] Tsai C.-L. et al., “Hyperspectral imaging combined with artificial intelligence in the early detection of esophageal cancer,” Cancers 13, 4593 (2021). 10.3390/cancers13184593 34572819 PMC8469506

[29] Tsai T.-J. et al., “Intelligent identification of early esophageal cancer by band-selective hyperspectral imaging,” Cancers 14, 4292 (2022). 10.3390/cancers14174292 36077827 PMC9454598

[30] Yoon J. et al., “A clinically translatable hyperspectral endoscopy (HySE) system for imaging the gastrointestinal tract,” Nat. Commun. 10, 1902 (2019). 10.1038/s41467-019-09484-4 31015458 PMC6478902

[31] Zhao Q. et al., “Heat treatment of human esophageal tissues: effect on esophageal cancer detection using oxygenated hemoglobin diffuse reflectance ratio,” Laser Phys. 21, 559–565 (2011). 10.1134/S1054660X11050380

[32] Transon J. et al., “Survey of hyperspectral earth observation applications from space in the sentinel-2 context,” Remote Sens. 10, 157 (2018). 10.3390/rs10020157

[33] Hosoe N. et al., “Capsule endoscopy for small-intestinal disorders: current status,” Digest. Endosc. 31, 498–507 (2019). 10.1111/den.13346 30656743

[r34] Iddan G. et al., “Wireless capsule endoscopy,” World J. Gastroenterol. 405, 417–417 (2000). 10.3748/wjg.14.1969 10839527

[r35] Niikura R. et al., “Associations between drugs and small-bowel mucosal bleeding: multicenter capsule-endoscopy study,” Digest. Endosc. 30, 79–89 (2018). 10.1111/den.12922 28719079

[36] Park S. et al., “Capsule endoscopy to detect normally positioned duodenal papilla: performance comparison of SB and SB2,” Gastroenterol. Res. Pract. 2012, 202935 (2012). 10.1155/2012/202935 22548051 PMC3324898

