# Supplementary material for: Small intestinal bleeding prediction by spectral reconstruction through band selection
Source: J Biomed Opt. 2025 Mar 19;30(3):036004. doi: 10.1117/1.JBO.30.3.036004 (PMC11922165; doi:10.1117/1.JBO.30.3.036004)
Supplement: Supplementary file 1 [file JBO_030_036004_SD001.pdf]

Supplemental Material:

Hsin-Yu Kuo [a], Riya Karmakar [b], Arvind Mukundan [b], Chu-Kuang Chou [c,d], Tsung-Hsien Chen [e], Chien-Wei Huang [f,g], Kai-Yao Yang [f,*], and Hsiang-Chen Wang [b,h,i,*]

[a] *Department of Internal Medicine, National Cheng Kung University Hospital, College of Medicine, National Cheng Kung University, No.1, University Road, Tainan City 701, Taiwan. Telomere-aging@hotmail.com.tw (H.-Y.K.)*
[b] *Department of Mechanical Engineering, National Chung Cheng University, 168, University Rd., Min Hsiung, Chia Yi 62102, Taiwan; karmakarriya345@gmail.com (R.K.); d09420003@ccu.edu.tw (A.M.)*
[c] *Division of Gastroenterology and Hepatology, Department of Internal Medicine, Ditmanson Medical Foundation Chia-Yi Christian Hospital, Chiayi 60002, Taiwan; vacinu@gmail.com (C.-K.C.)*
[d] *Obesity center, Ditmanson Medical Foundation Chia-Yi Christian Hospital, Chiayi 60002, Taiwan*
[e] *Department of Internal Medicine, Ditmanson Medical Foundation Chia-Yi Christian Hospital, Chiayi 60002, Taiwan. cych13794@gmail.com (T.-H.C.)*
[f] *Department of Gastroenterology, Kaohsiung Armed Forces General Hospital, 2, Zhongzheng 1st.Rd., Lingya District, Kaohsiung City 80284, Taiwan. yangkaiyao@gmail.com (K.Y.Y.); forevershiningfy@yahoo.com.tw (C.-W.H.)*
[g] *Department of Nursing, Tajen University, 20, Weixin Rd., Yanpu Township, Pingtung County 90741, Taiwan*
[h] *Director of Technology Development, Hitspectra Intelligent Technology Co., Ltd., Kaohsiung 80661, Taiwan*
[i] *Department of Medical Research, Dalin Tzu Chi Hospital, Buddhist Tzu Chi Medical Foundation, No. 2, Minsheng Road, Dalin, Chiayi, 62247 Taiwan*

1 VGG-16

The VGG architecture was introduced in 2014 by the Visual Geometry Group (VGG) at the University of Oxford's Department of Science and Engineering. The primary objective is to demonstrate the impact of increasing the depth of a network on its final performance, up to a given degree. The VGG architecture consists of two distinct components, known as VGG16 and VGG19. With the exception of variations in network depths, there exists no fundamental distinction between them. In contrast to AlexNet, the VGG architecture introduced in 2012 emphasizes the utilization of a significant quantity of 3 x 3 convolutions. The author posits that substituting larger convolutions with smaller convolutions has the potential to enhance the quantity of information. Furthermore, the utilization of smaller convolutional layers might enhance nonlinearity and decrease the number of parameters in comparison to larger convolutional layers.

The VGG architecture incorporates distinct processing techniques for the training and validation datasets, with the training phase employing multi-level training methodologies. During each training iteration, a random number is taken from a predetermined range of random integers. This number is then scaled accordingly and afterwards cropped to the required size in a random manner. In the verification phase, the prediction process involves the utilization of several cropping blocks. The data is resized and divided into segments, namely the upper left, upper right, lower left, lower right, and middle sections. Each segment is predicted using a predetermined cropping size. Finally, the projected results from all segments are averaged to obtain the final prediction outcome. Figure 1 represents the architecture.

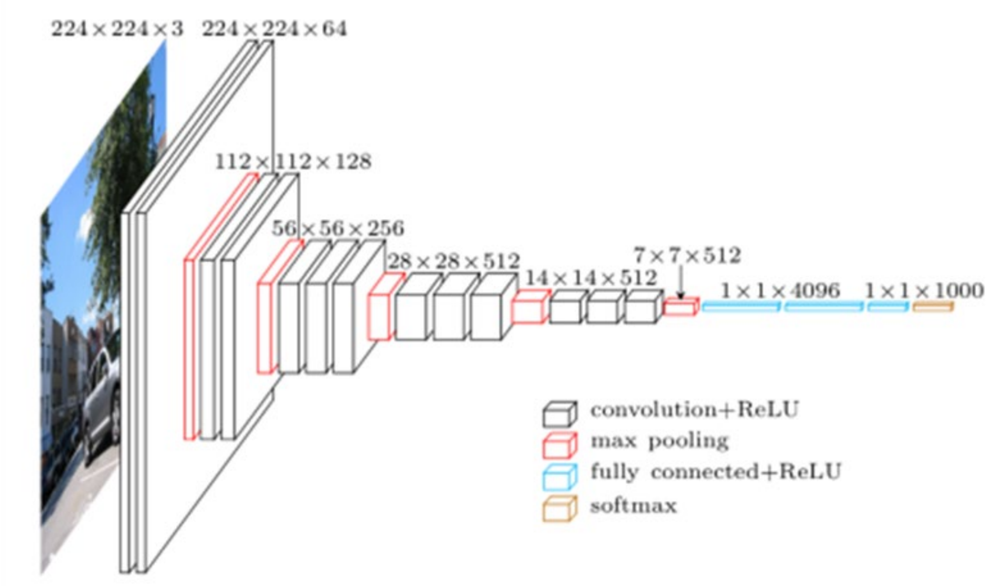

Figure S1: VGG-16 architecture diagram

2 Confusion matrix

A commonly employed evaluation technique in deep learning involves tabulating the actual and predicted numbers for correct and incorrect classifications. This method divides the data into four categories: True Positive (TP), False Positive (FP), False Negative (FN), and True Negative (TN). The evaluation is then conducted by analyzing the relative proportions of these categories. Table 1 is a schematic diagram of the binary classification confusion matrix. The following are the evaluation criteria used in this study, and the prediction of disease is the specific criterion:

Accuracy is the most common standard. It is based on the proportion of "correctly predicted" in the "total number of patients", representing the proportion of patients who are correctly predicted to be sick or not sick among all patients. The formula is represented in equation (1)

Accuracy=(TP+TN)/ (Total Number) .... (1)

Precision refers to the ratio of accurately predicted instances inside the set of expected sick patients, indicating the proportion of forecasted sick patients among all the predicted sick patients. This method can be employed to assess the extent to which hospital resources are utilized by this disease. If the threshold is set too low, it can result in a significant number of patients being erroneously diagnosed as ill, leading to a considerable waste of medical resources. The formula is represented in equation (2).

Precision=TP/ (TP+FP) .... (2)

Recall (Sensitivity) is based on the proportion of "correctly predicted" among the "real sick patients", representing the proportion of predicted sick patients among the actual sick patients. For diseases, this indicator is relatively important. The higher it is, it means that very few patients who are sick will be regarded as disease-free, which will not cause the disease to continue to worsen and achieve the purpose of early treatment. The formula is represented in equation (3).

Recall=TP/ (TP+FN) .... (3)

Specificity is the proportion of "correctly predicted" patients among "actually disease-free patients", representing the proportion of predicted disease-free patients among the actual disease-free patients. It has the same meaning as Precision but is just the opposite of Recall. It is aimed at patients without disease. The formula is represented in equation (4).

Specificity=TN/ (FP+PN) .... (4)

F1 Score is a combination of the two evaluation criteria of Precision and Recall, which can be expressed as an average of the two indicators. The formula is represented in equation (5).

$$F1\ Score=2/(1/Precision+1/Recall) \quad \ldots\ldots\ldots\ldots (5)$$

Table S1: Confusion matrix results of seven data sets

**WLI**		**Actual**		
		**bleed**	**unbleeded**	**Total**
**predict**	**bleed**	95	19	114
	**unbleeded**	26	126	152
	**Total**	121	145	266
**540~780(nm)**		**Actual**		
		**bleed**	**unbleeded**	**Total**
**Predict**	**bleed**	57	27	84
	**unbleeded**	64	118	182
	**Total**	121	145	266
**470~500+540~780(nm)**		**Actual**		
		**bleed**	**unbleeded**	**Total**
**Predict**	**bleed**	37	6	43
	**unbleeded**	84	139	223
	**Total**	121	145	266
**470~500(nm)**		**Actual**		
		**bleed**	**unbleeded**	**Total**
**Predict**	**bleed**	72	25	97
	**unbleeded**	49	120	169
	**Total**	121	145	266
**405~415+470~500+540~780(nm)**		**Actual**		
		**bleed**	**unbleeded**	**Total**
**Predict**	**bleed**	94	43	137
	**unbleeded**	27	102	129
	**Total**	121	145	266
**405~415(nm)**		**Actual**		
		**bleed**	**unbleeded**	**Total**
**Predict**	**bleed**	97	8	105
	**unbleeded**	24	137	161
	**Total**	121	145	266
**405~415+535~545(nm)**		**Actual**		
		**bleed**	**unbleeded**	**Total**

**Predict**	**bleed**	69	32	101
	**unbleeded**	52	113	165
	**Total**	121	145	266

3. Hyperspectral Results

S.no	Before Camera Calibration	Spectrometer	Chromatic Aberration	After Camera Calibration	Spectrometer	Chromatic Aberration
1			7.08			1.24
2			7.63			0.78
3			16.43			0.86
4			12.45			1.68
5			14.92			0.45
6			10.80			0.05
7			7.47			0.52
8			18.46			0.22
9			13.19			0.62
10			8.09			1.30
11			8.03			0.09
12			6.43			0.58
13			10.32			0.30
14			12.19			0.23
15			13.31			0.17
16			7.00			0.18
17			17.80			0.03
18			22.22			0.19
19			0.00			0.08
20			5.30			0.30
21			9.77			0.42

22			12.71			0.81
23			13.34			2.01
24			3.37			1.96
Average Color Difference			10.76	Average Color Difference		0.63

Table S1. The color difference before and after camera calibration

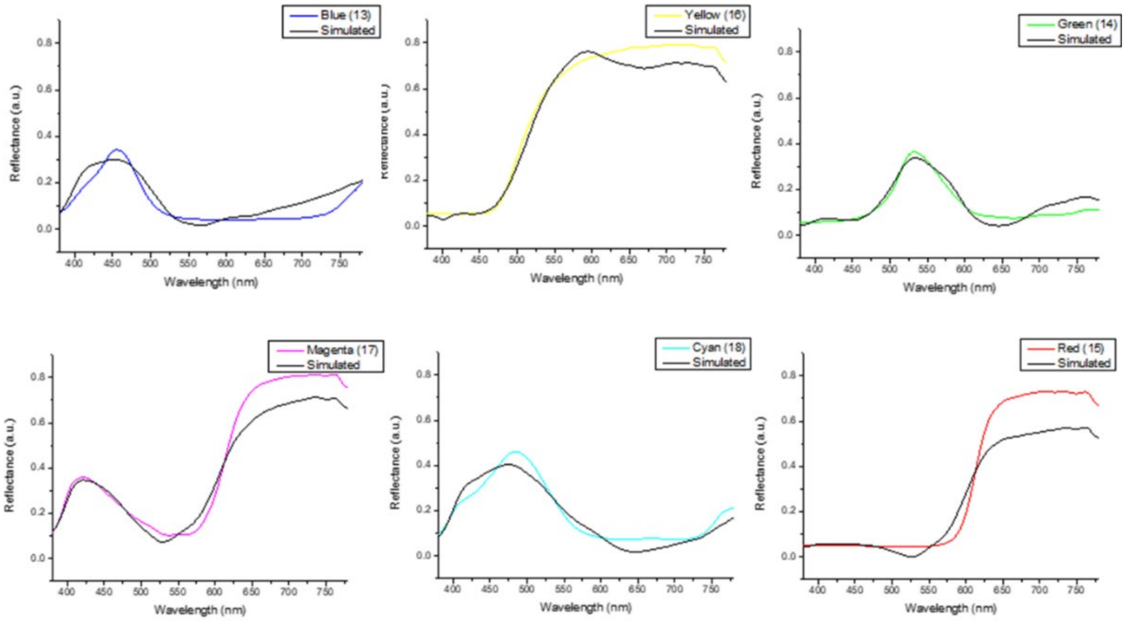

Figure S3. RMSEs between analog and measured spectra of each color block

Measured Color				Simulated Color				Color Difference
L	a	b	Color	L	a	b	Color	
37.61	13.65	24.56		37.64	11.87	22.60		1.32
66.48	14.68	31.10		66.37	15.47	30.58		0.73
50.44	-7.58	-6.44		50.72	-8.16	-6.09		0.75
42.80	-16.14	30.50		42.48	-14.04	31.19		1.50
56.16	5.70	-8.01		55.96	5.89	-8.51		0.43
70.99	-34.14	16.44		71.01	-34.00	15.65		0.41
61.97	32.36	66.76		61.85	33.07	65.32		0.80
40.20	6.07	-27.03		40.27	6.15	-26.67		0.27
51.59	46.04	27.52		51.72	44.60	27.21		0.52
30.62	18.70	-9.45		30.52	20.85	-8.70		1.46
72.24	-24.91	66.55		72.23	-25.21	66.79		0.13
72.46	17.04	75.67		72.59	16.33	76.17		0.49
29.18	13.90	-37.66		28.70	15.23	-38.14		0.81
55.59	-40.93	42.88		55.56	-41.82	42.54		0.42
41.66	53.78	34.95		41.60	54.34	34.25		0.51
82.26	1.48	87.73		82.21	1.96	87.67		0.27
51.29	46.36	1.08		51.30	46.12	0.95		0.10
50.80	-31.41	-12.85		50.66	-30.83	-13.20		0.42
95.47	-3.88	21.64		95.38	-3.70	22.54		0.52
80.96	-3.08	18.47		81.32	-3.39	17.74		0.63
66.38	-2.74	15.56		66.33	-3.33	15.44		0.69
52.18	-2.26	12.86		51.36	-2.59	12.85		0.91
36.47	-2.05	9.55		37.78	-3.18	8.64		2.00
21.40	-1.45	6.28		20.70	-2.87	7.50		2.02
							Average Color Difference	0. 75

Figure S4. LAB values of the simulated and observed colors.
